# Accuracy of 3D printing of the samples in the prototyping of cavity-based metamaterials

**DOI:** 10.1038/s41598-025-98391-4

**Published:** 2025-04-17

**Authors:** Bartłomiej Chojnacki, Aleksandra Chojak, Wojciech Binek, Jan Pawlik, Julia Idczak

**Affiliations:** AGH University of Kraków, Mickiewicza Av. 30, Cracow, 30-059 Poland

**Keywords:** Mechanical engineering, Design, synthesis and processing, Metamaterials, Mechanical properties

## Abstract

Recent developments in designing cavity-based metamaterials require advanced methods for prototyping measurement samples, such as 3D printing techniques, which allow for the manufacture of complicated shapes unavailable for other technologies. However, the imperfections of the printouts, which vary for different printing technologies and printer settings, may cause differences between the designed and measured acoustic performance of the samples. This paper presents a complex study of the influence of printing details on the resulting sound absorption coefficient of a simple cavity-based metamaterial consisting of a coiled-up Helmholtz resonator. A general approach for manufacturing measurement samples is discussed. It is shown that the samples should be printed in one whenever possible, and joining the parts results in the formation of microslits and leads to mismatching of the sample impedance. Different printing technologies were also compared (SLS, DLP, FDM), and it was shown that for the studied geometry, the differences obtained for different printers are negligible from a practical point of view. The settings of the FDM printer were also studied for comparison with previous works by other authors. It was shown that the printer settings and the choice of filament are less important than random printing errors, which may occur regardless of the printer settings.

## Introduction

Acoustic metamaterials are artificial structures typically designed for a particular function, such as sound absorption^1–3^, sound diffusion^4,5^, or sound insulation^6,7^. The main reason for using metamaterials is their better performance compared to that of conventional materials: reduced thickness, improved acoustic parameters, or both. Recent developments in designing acoustic metamaterials require advanced manufacturing techniques, especially 3D printing, for both prototyping and application purposes. Printed elements are broadly used in different types of acoustic metamaterials, including vibroacoustic spring-mass resonators, cavity-based metamaterials, and even membrane-type resonators^3,8–10^. In the case of cavity-based metamaterials, the printing process is not generally considered a problem; however, some authors acknowledge that the acoustic properties of the samples differ with different 3D printing technologies and printing parameters and present different acoustic properties than previously indicated by modeling results^11–14^. The three most widely used methods for 3D printing are fused deposition modeling (FDM), direct light projection (DLP), and selective laser sintering (SLS). A common feature of these techniques is that the elements are printed layer by layer; however, the production of layers differs depending on the method. The first method (FDM) relies on the deposition of a molten filament extruded from a heated nozzle driven along a numerically controlled path. The main advantages of this method are its cost-effectiveness, availability, good community support, and usually, very little postprocessing. On the other hand, the printout resolution and surface quality are usually worse than those of the other mentioned methods. Overhanging surfaces require a support structure or well-calibrated bridging parameters. The DLP method involves projecting UV light (wavelength 365–405 nm) onto a vat of UV-sensitive resin. A digitally controlled liquid crystal mask selectively blocks or allows the light to pass through, shaping it into a desired pattern, resulting in a very thin but continuous layer of the printout. This technology is also widely available and inexpensive. It provides excellent printout resolution and pixel-scale precision. The greatest drawbacks of DLP are problematic postprocessing, moderate toxicity of the resin fumes in poorly ventilated areas, and the occurrence of stress concentrations in large flat areas. Postprocessing also requires draining the residual liquid resin from the model cavities before the final curing of the printout. The SLS method relies on a laser that fuses powdered material in subsequent layers, resulting in durable, complex, and precise printouts. SLS printouts are usually the strongest and most durable. Due to minimal anisotropy, the durability of the printouts is comparable to that of the injection molding. In this technique, supports are not required since the printing process occurs in a compartment filled with tightly packed powder; however, in the case of complex internal channels within the model, cleaning of the residue powder is difficult. The surface quality of the proposed method is superior to that of the FDM method, but models printed with DLP printers still yield better results.

Usually, the observed values of the sound absorption coefficient of the printouts increase in comparison with those of analytical models. The low roughness caused by the layers, particularly in the FDM-printed samples, results in a sound absorption coefficient that increases the resulting sound absorption of the unit cell. The resulting roughness of the walls was mainly attributed to the layer height, which is one of the printing parameters^12^. In another paper by the same authors, it was shown that changing other printing parameters, such as reducing printing speed, may improve the quality of the printout but still does not resolve the issue of excessive sound absorption by the sample^15^. Different authors have shown that the acoustic performance of FDM-printed coiled-up resonators depends on variations in their geometrical parameters, which are easily influenced by thermal conditions, resulting in a frequency shift of the main peaks^16^. Other printing parameters were investigated in the study by Fusaro et al.^17^. The authors confirmed the results presented by Ciochon et al. and provided more extensive practical analyses and results, showing that the acoustic performance of the structures also depends on the infill parameters and the type of filament used. The FDM technique, along with the use of PET-G filaments, was indicated as the optimal choice for manufacturing cavity-based metamaterials with coiled-up resonators.

This paper presents a complex study on the manufacturing approach, 3D printing techniques and parameters, and their impact on the resulting sound absorption coefficient of a coiled-up resonator. Compared to the other studies, in this case, the authors focused on the magnitude of the negative impact of possible fabrication inconformities occurring in certain corner cases of the additive manufacturing process. Instead of focusing on particular printing parameters or types of filaments, more general issues are considered. Three particular topics are discussed: the general approach for printing and sealing metamaterials with coiled-up resonators, the choice of 3D printing technique (FDM, SLS, DLP), and the parameters of the FDM settings to complement the previously mentioned studies. In this research, we prove that the possible influence of the printing setup may not be as crucial as previously shown in the state-of-the-art, and postprint quality control is the key factor. By maintaining control over the printing process and avoiding typical print faults, it is possible to obtain fully functioning samples, even with a coarse printing setup, regardless of the type of filament material or the type of printer.

**Fig. 1:**
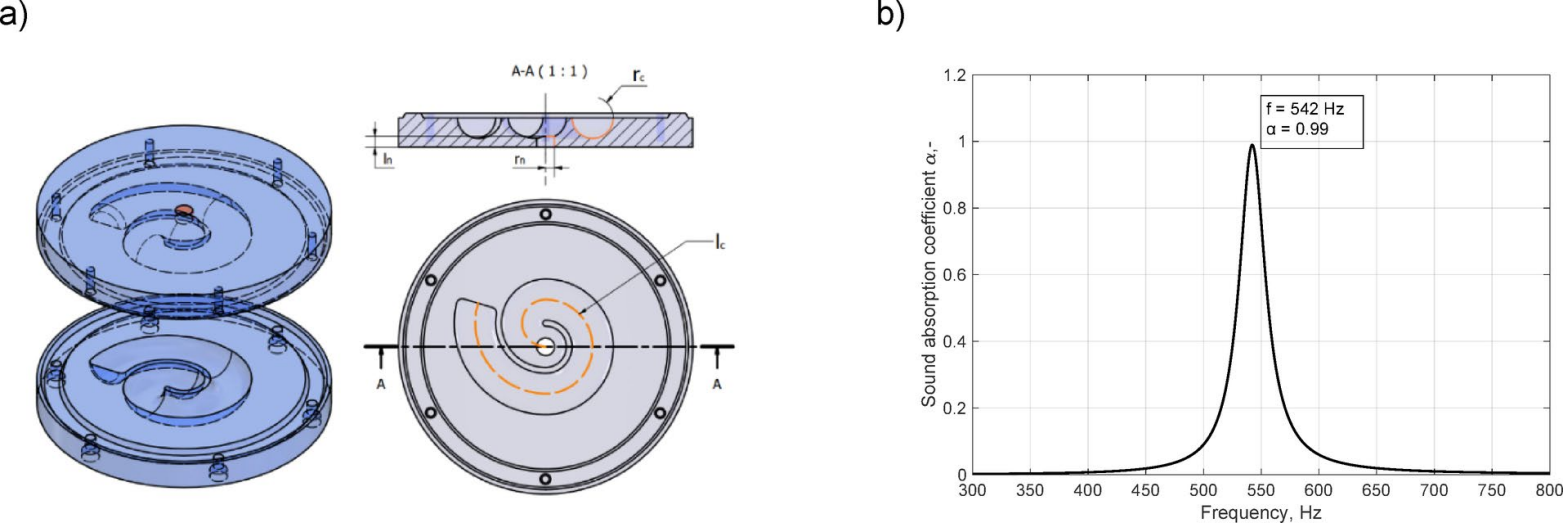
(a) Geometry of the samples manufactured in the study and (b) the modeled sound absorption coefficient (finite element model).

The choice of geometry for this particular study was based on previously reported research. To compare the results with those of other authors, a coiled-up Helmholtz resonator was chosen, a particular type of cavity-based metamaterial, similar to what was previously used by Huang^9^. The resonance frequency was adjusted to ~ 540 Hz to introduce changes to the canal, such as increasing the distance between coiled parts of the canal (increasing the thickness of the shell of the canal). The geometry of the sample is shown in Fig. 1a, and the modeled sound absorption coefficient is shown in Fig. 1b. A perfect sound absorption (sound absorption coefficient $\:\alpha\:=0.99$) can be observed at 542 Hz. The neck and cavity of the Helmholtz resonator are both circular, the radius of the neck is 3 mm, the length of the neck is 3 mm, the radius of the cavity canal is 7 mm, and the length of the canal is 120 mm. The wall of the canal (shell) is 2 mm thick. The diameter of the unit cell corresponds to the diameter of the B&K 4206 impedance tube and is equal to 100 mm.

## Results

### Selection of the 3D printing technique and sealing

**Fig. 2:**
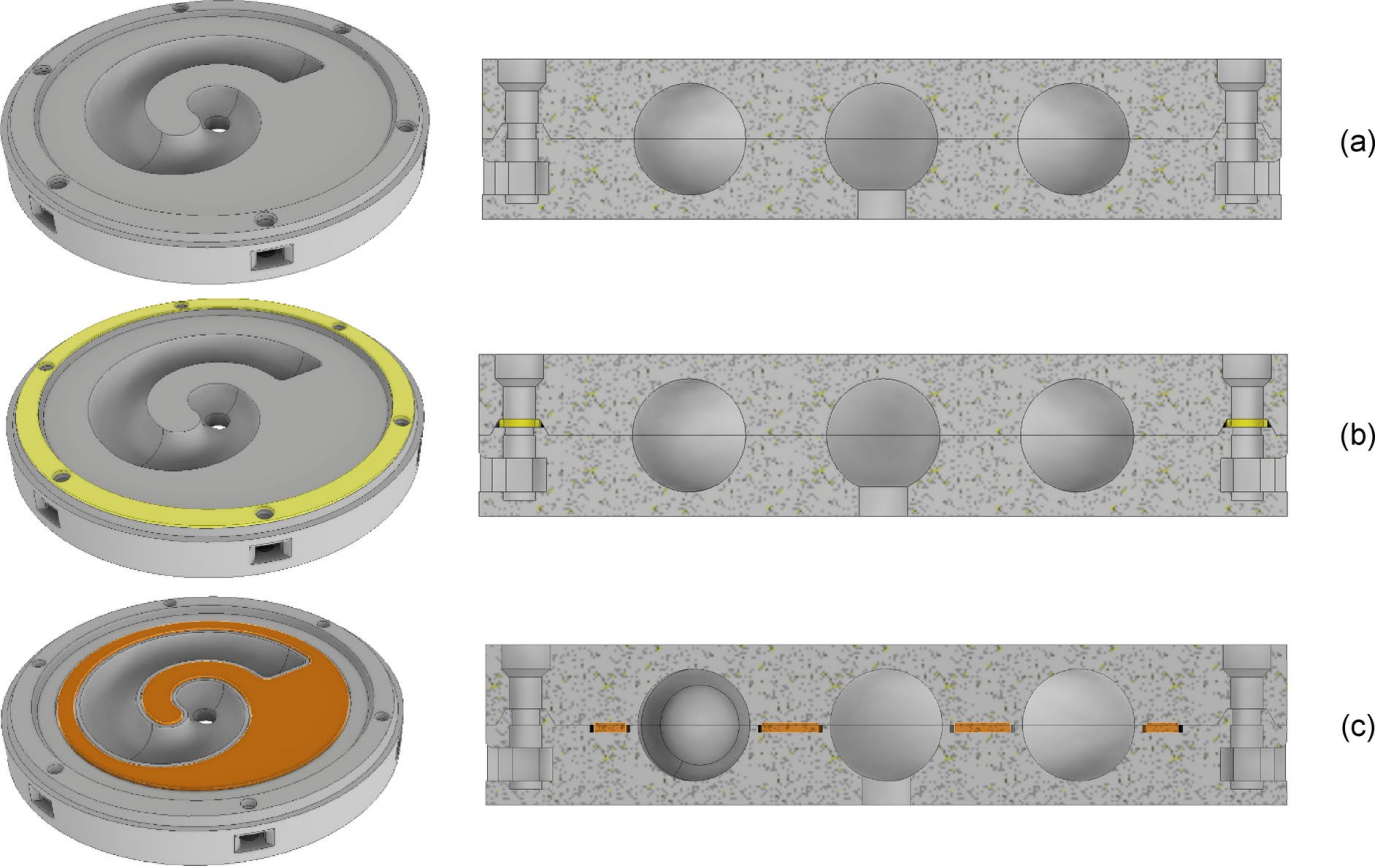
Types of sealings used in the study, (a) no additional sealing, (b) ring seal, (c) shaped seal. The rectangular pockets are M3 nut slots.

In the case of a coiled-up resonator, the first idea was to print the measurement sample in two halves and then join them together. This allows for a visual inspection of the print quality of the inner walls for the samples 100 mm in diameter and a canal with a 7 mm radius. The splitting was also coerced by the specific limitations of powder- and resin-based additive manufacturing technologies. Hypothetically, one could print such a resonator as one integral part; nevertheless, it would be difficult to estimate the uncured or unsintered material residues inside the canal. Additionally, in the case of FDM-printed samples, splitting helped to find the correct range of parameters (such as printing speed, retraction length, and retraction speed) for the utilized material and diminish the sagging of overhanging surfaces of the resonator canal. The issue of joining the halves of the samples has not been discussed in the previously mentioned research by the other authors, where the samples were always printed in one piece—the size of the sample and the canals in the study by Fusaro et al.^17^ (39 mm) allowed for a uniform printout, and in the case of Zielinski et al.^13^, the studied geometry would not benefit from a different printing orientation or from dividing the sample into smaller parts. The original design (and the computer model) of a coiled-up resonator, however, includes no connection between the halves of the measurement sample; therefore, they need to be perfectly sealed. The halves were always joined together with a set of six M3 screws located on the circumference, fastened with M3 nuts positioned and locked in place in specifically designed pockets on the lateral side of the cylinder. Figure 2 depicts the three different sealing approaches under study:

- No seal (flat-to-flat surface with embossed rim for positioning);
- Ring seal,
- A shaped seal.

**Fig. 3:**
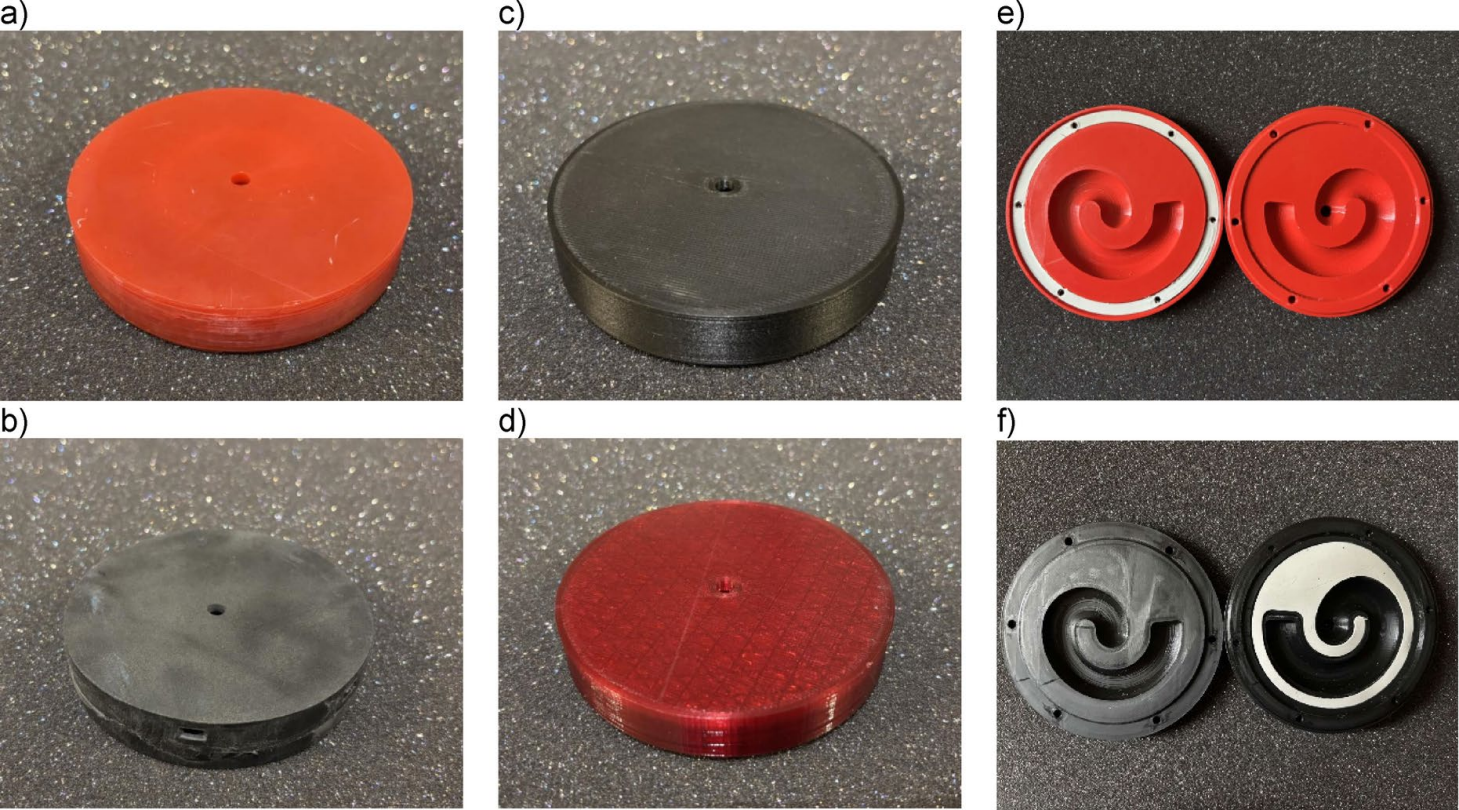
Pictures of the example samples and their masses after manufacturing: (a) DLP (160 g), (b) SLS (135 g), (c) FDM: PLA (76 g), (d) FDM: PET-G (78 g), (e) FDM-PLA with a ring seal (94 g), and (f) FDM-PLA with a shaped seal (96 g).

Both the ring seal and the shaped seal were fabricated with FDM technology with a TPU filament 40D on a Shore hardness scale. All the sealings were tested for all the available printing techniques (FDM – with PLA and PET-G filament, DLP, and SLS) and printed with the best possible dimensional accuracy (see Methods section for printing details). Several examples of the samples used in the present study are shown in Fig. 3. The caption of Fig. 3 includes the measured weight of the samples after manufacturing and assembly with all the required seals, as it is likely that the mass change between the samples could affect the performance of the metamaterial, i.e., it could lower the resonance frequency. However, such behavior was not observed in the present study.

**Fig. 4:**
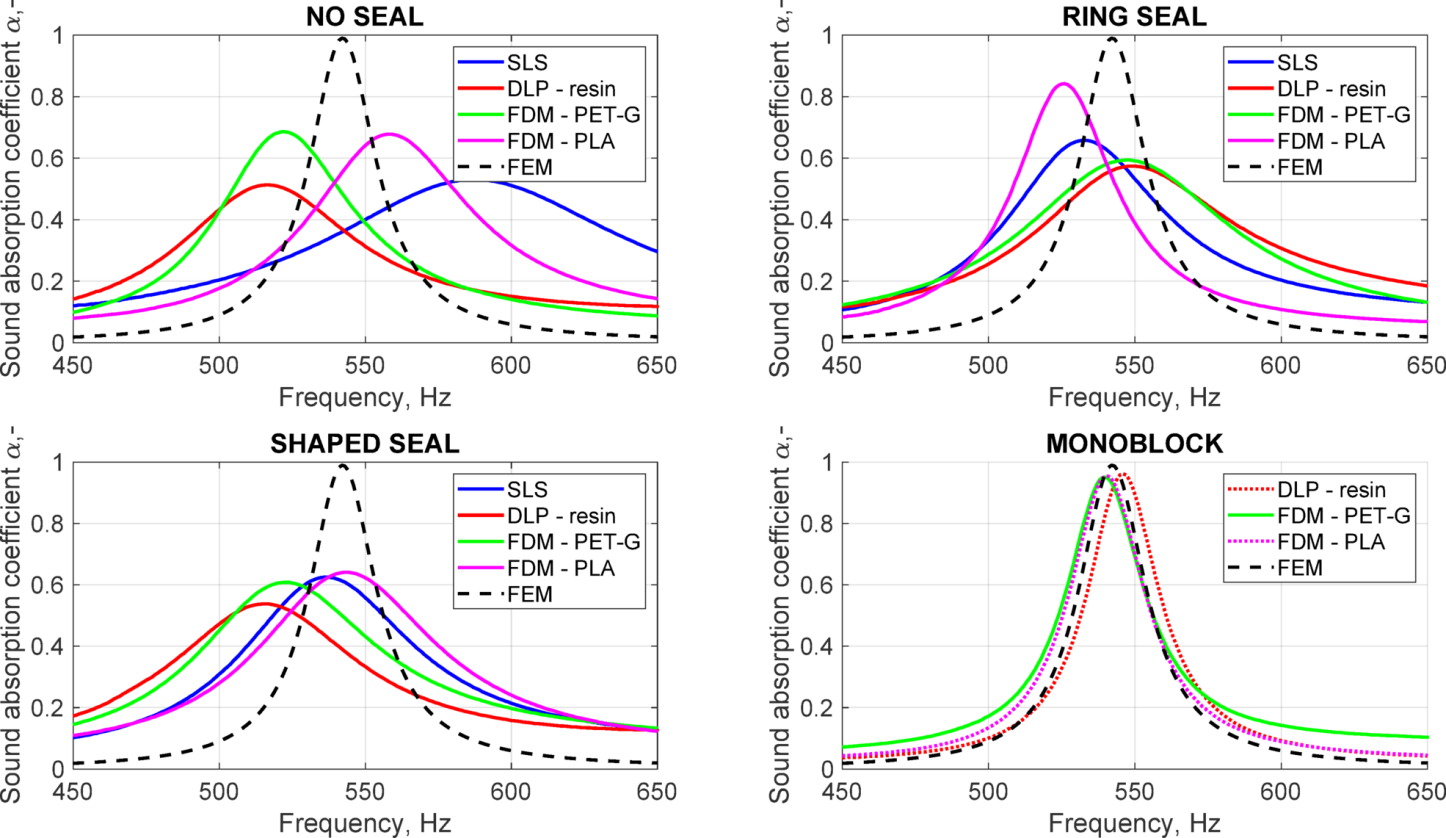
The measured sound absorption coefficient depending on the sealing method and printing technique.

In Fig. 4, we can observe large discrepancies between the measurement results obtained for the measurement samples printed in half and the expected sound absorption coefficient. Differences are observed both in terms of the resonance frequency and maximum sound absorption coefficient, regardless of the printing technology. This can be explained by the additional sound absorption caused by the imperfect sealing of the samples, which results in mismatching of the sample’s impedance. Additional sound absorption causes shifting of the zero pole pair in the complex frequency plane and results in a decreased maximum sound absorption coefficient, shifted resonant frequency, and decreased quality factor of the resonance. Notably, neither the printing technology nor the sealing method significantly impacts the obtained results. The only measurement results comparable with the modeling results are those obtained for the samples printed in one. There is no sample printed in one with the SLS method since this particular technology would not allow for removing the remaining material from the inside of the resonator. The resonant frequencies obtained for both the FDM and DLP samples are 540 Hz and 546 Hz, respectively (0.4% lower and 0.7% higher than the modeling result). The maximum measured sound absorption coefficient is 0.96 for all the samples (3% less than the modeling results). This accuracy of the printouts can be considered sufficient for practical applications. These small differences can be attributed to the low roughness of the inner walls of the canals, and practical applications can be neglected. It is important to note that the FDM and DLP result in similar printout accuracies. In such a situation, the accessibility and price of the FDM technique make it more advantageous than the DLP technique, especially for prototyping purposes.

### FDM parameter settings

In the previous section, it was shown that the FDM is the most advantageous of all the available techniques for the geometrical case under study. The question is how precise the printouts need to be to obtain satisfactory results. The authors of the previous studies focused mainly on the printing speed, layer height, and infill type and density. Differences were shown between the infill ratios, filaments, and printout settings. Fusaro et al.^17^ claimed that the best possible set of parameters relies on the proper combination of infill percentage, printing speed, and layer height. However, in the present study, the printing speeds were greater, and the infill percentage was lower than those in the study by Fusaro et al. Intuitively, these conditions should result in worse quality measurement samples in terms of acoustic parameters. However, importantly, the other authors did not consider shell thickness. In the study by Fusaro et al., the shell thickness varied between the top and bottom shell thicknesses (0.48–0.6 mm) and between the top and bottom shell thicknesses (1.6 mm); in the present study, the specimens were fabricated with a constant shell thickness of 2 mm. A comparison of the printout parameters between the study by Fusaro et al. and the present study is shown in Table 1.

**Table 1:** Comparison of the printout parameters between the study by Fusaro et al.^17^ and the present study.

	Fusaro et al.				Current study			
	“Fast Coarse”	“Fast Fine”	“Slow Coarse”	“Slow Fine”	dn 0.4, lh 0.2	dn 0.4, lh 0.3	dn 0.5, lh 0.2	dn 0.5, lh 0.3
Parameter	F_FCoarse	F_FFine	F_SCoarse	F_SFine	L_4 − 2	L_4 − 3	L_5 − 2	L_5 − 3
Nozzle diameter	0.4	0.4	0.4	0.4	0.4	0.4	0.5	0.5
Layer height	0.30	0.12	0.30	0.12	0.2	0.3	0.2	0.3
Top/Bottom Layers	2	4	2	4	10	7	10	7
# perimeters	4	4	4	4	5	5	4	4
Shell thickness	0.6–1.6	0.48–1.6	0.6–1.6	0.48–1.6	2	2	2	2
Base speed	80	80	50	50	80	80	80	80
Outline speed	25	25	15	15	40	40	40	40
Solid speed	50	50	30	30	40	40	40	40
Infill speed	75	75	45	45	80	80	80	80
Infill %	30	30	30	10–50	20	20	20	20
Infill type	Gyroid	Gyroid	Gyroid	Gyroid	Stars	Stars	Stars	Stars

In the study by Ciochoń et al.^18^, the only printing parameter under investigation was the layer height. It was chosen to be the most important factor influencing the surface roughness of the printout walls. The considered layer heights were 0.1, 0.15, 0.2, and 0.25 mm, while in the present study, the minimum layer height was 0.2. It must be noted that the type of metamaterial under study was different from that described in the present study; however, it was shown that the maximum sound absorption coefficient is significantly influenced by the resulting surface roughness. Zieliński et al.^19^ provided information only on the type of 3D printing technique and lacked details on the printer settings; therefore, a detailed comparison with this study is impossible. An inspection of the printout parameters used in the present study could provide guidance, particularly in comparison with the findings of the study by Fusaro et al.^17^; however, as 3D printing is a complex and multiparametric process, these results should not be attributed only to the combination of printing speed and layer height.

**Fig. 5:**
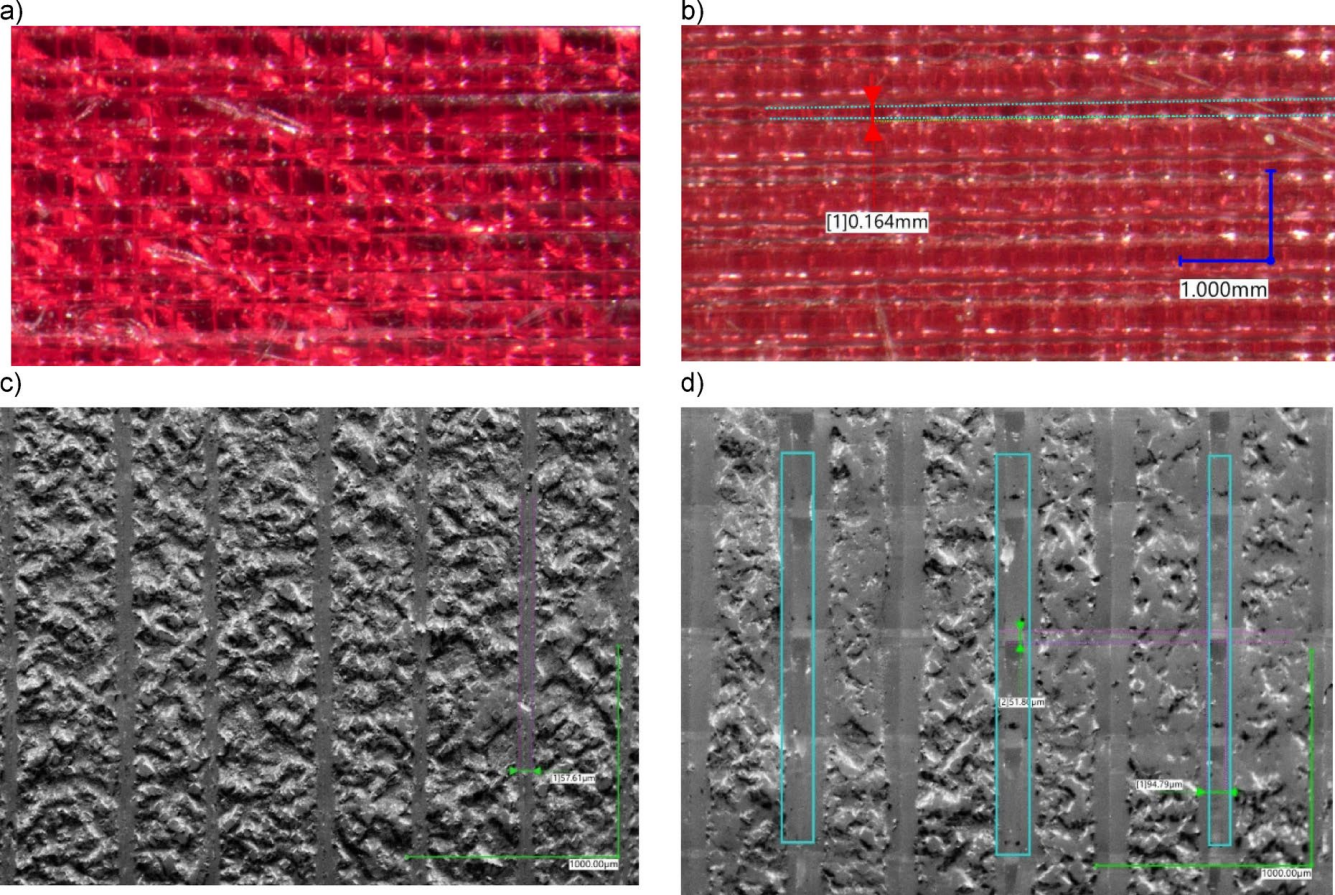
Surface printed with two different temperature settings: (a) 5° higher than recommended by the filament manufacturer, (b) according to the recommendations; unintended air gap is marked, (c) the grey-scale representation of the properly printed sample surface with the layer-size marked as 57.61 μm, (d) grey-scale micro-slits marked that occurred in the internal volume on the surface, below the solid surface.

The FDM process relies on fusing molten strings of thermoplastics with circular cross-sections; therefore, some air gaps between cross-stacked strings may form. Figure 5 shows a microscopic image of the 3D-printed surface of a preliminary sample. Figure 5a shows a sample with a printing temperature 5 °C higher than that recommended by the manufacturer, and the sample shown in Fig. 5b was printed at the recommended temperature and represents unintended air gaps invisible to the naked eye. The proper melting temperature may vary between printers, as the thermistors or thermocouples are usually installed “as is” without additional calibration. The air gaps between lines and layers can be diminished by adding more solid layers, increasing the infill density, lowering the speed, or adjusting the flow rate percentage. However, the formation of air gaps might also depend on other parameters, such as the number of perimeters (especially considering channels with circular cross-sections).

In this study, two different filaments, PLA (polylactic acid) and PET-G (polyethylene terephthalate with glycol admixture), were tested for four combinations of nozzle diameter (dn) and layer height (lh). These materials are commonly used in 3D printing due to their ease of use and low toxicity. PLA is generally considered safe for use due to its biodegradability, while PET-G can be recycled. While the exact physical properties tend to vary between particular products and manufacturers, the parts printed with PLA tend to be more rigid and brittle, and parts fabricated with PET-G are usually more flexible^20,21^. Both filaments exhibit good interlayer adhesion and have similar densities—1.23 g/cm^3^ for PLA and 1.27 g/cm^3^ for PET-G.

The samples were manufactured with two nozzle diameters: 0.4 mm (standard nozzle) and 0.5 mm. The choice of nozzle determined the minimal and maximal layer heights. According to the rule of thumb, the layer height should be within 0.2 and 0.75 of the nozzle diameter; therefore, the lowest layer height was 0.2 mm, and the highest layer height was 0.3 mm. Every set of settings was adjusted to obtain horizontal and vertical shell thicknesses possibly close to 2 mm; for instance, for a 0.5 mm nozzle and 0.3 mm layer height, the sample had 4 perimeters (4 × 0.5 mm = 2 mm) and 7 top and bottom solid layers (0.3 × 7 = 2.1 mm).

**Fig. 6:**
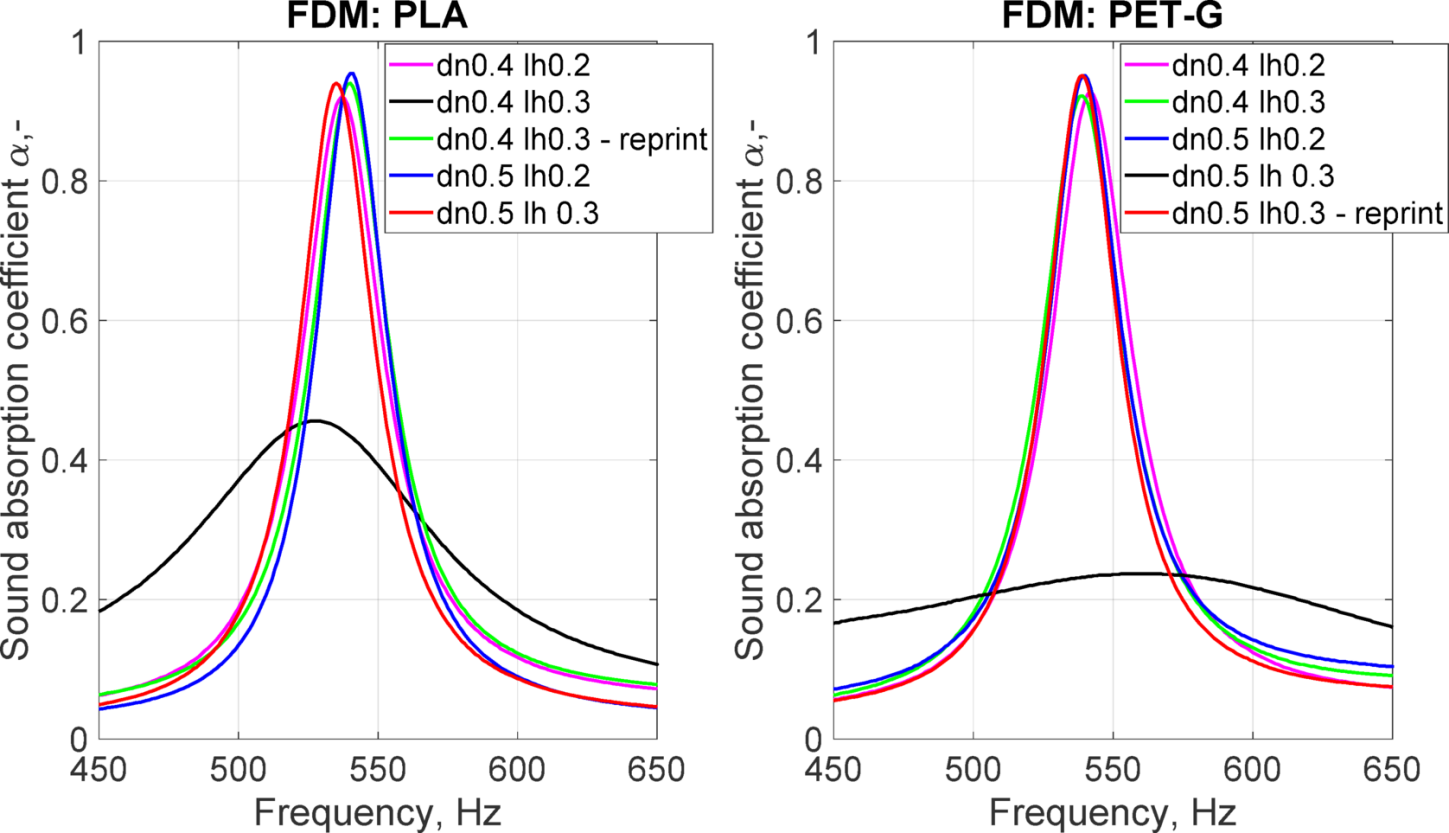
The sound absorption coefficient depending on the FDM printing parameters; dn is the diameter of the printer nozzle (mm), and lh is the layer height (mm).

For both tested filaments, PLA and PET-G, we can observe one outlying set of results (Fig. 6): the maximum sound absorption coefficient decreases drastically, as does the quality factor of the resonance. In the case of PLA, this was observed for a nozzle diameter of 0.4 mm and a layer height of 0.3 mm. Such a result could be expected since this combination of nozzle diameter and layer height is at the upper limit for the recommendations (0.3 = 0.75 × 0.4)^22,23^. However, in the case of PET-G, outlying results were observed for the combination of a 0.5 mm nozzle diameter and 0.3 mm layer height, which is within the recommended range. Visual inspection of the samples did not reveal any obvious defects. The samples were reprinted with the same printing settings, and the obtained results matched the remaining configurations. For the PLA filament, the resonance frequencies varied between 535 and 540 Hz, and the maximum sound absorption was between 0.92 and 0.95. For PET-G, the resonance frequencies were within the range of 539–542 Hz, and the maximum sound absorption was between 0.92 and 0.95. Such differences are negligible for practical applications. This observation is crucial because this means that there are several other factors, independent of the printing parameters, that may cause defects to be impossible to observe via visual inspection, causing overall deterioration of the sample performance. The walls of the canal were, in any case, 2 mm thick, which means that at least four layers of filament were needed (in the case of a nozzle diameter of 0.5 mm) to represent the modeled geometry in a printout. This was thought to be enough to ensure that there was no connection between the coiled parts of the canal. However, random events during the printing process, such as filament inconsistency, filament spool inertia, extruder slip or even air bubbles trapped in the feed material during the manufacturing process, may have caused ruptures in the inner walls of the canal, which are impossible to detect when the sample is printed as one part^24^. Additionally, even slightly backlashed linear drives combined with high-axis acceleration may result in unexpected errors on the printout surfaces (Fig. 5b).

### Raptured walls of the canal

**Fig. 7:**
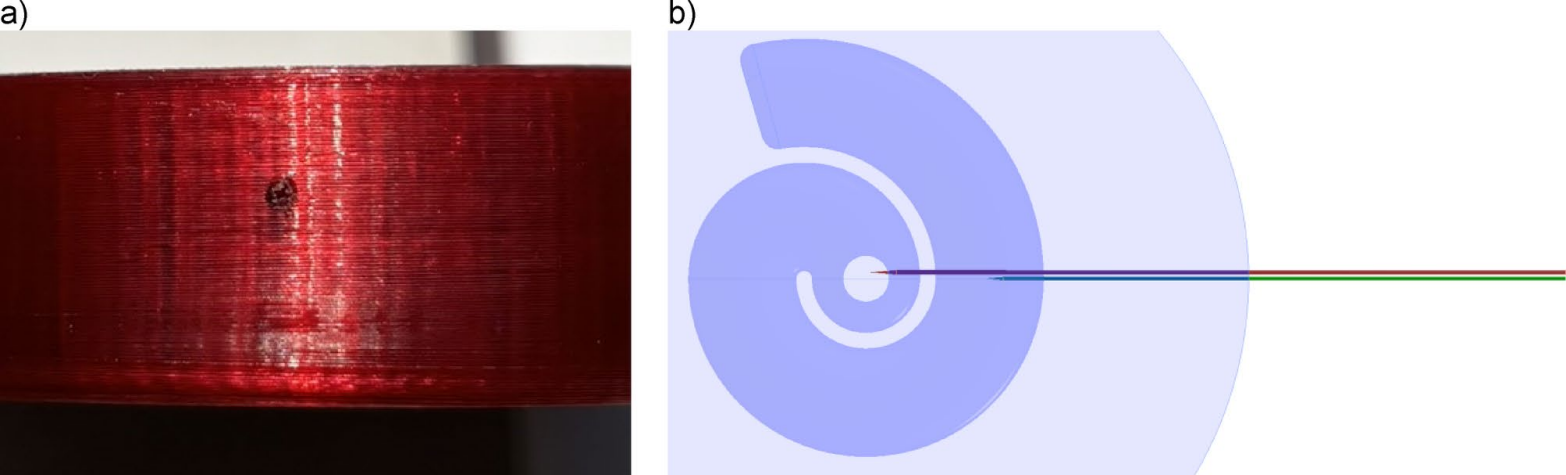
A hole made with a 0.6 mm tungsten wire: (a) inlet of the hole, (b) path of the rupture connecting the coiled parts of the canal, where green indicates shallow puncture and red indicates deeper puncture, connecting the coiled parts of the canal. In reality, both punctures shared the same inlet hole.

**Fig. 8:**
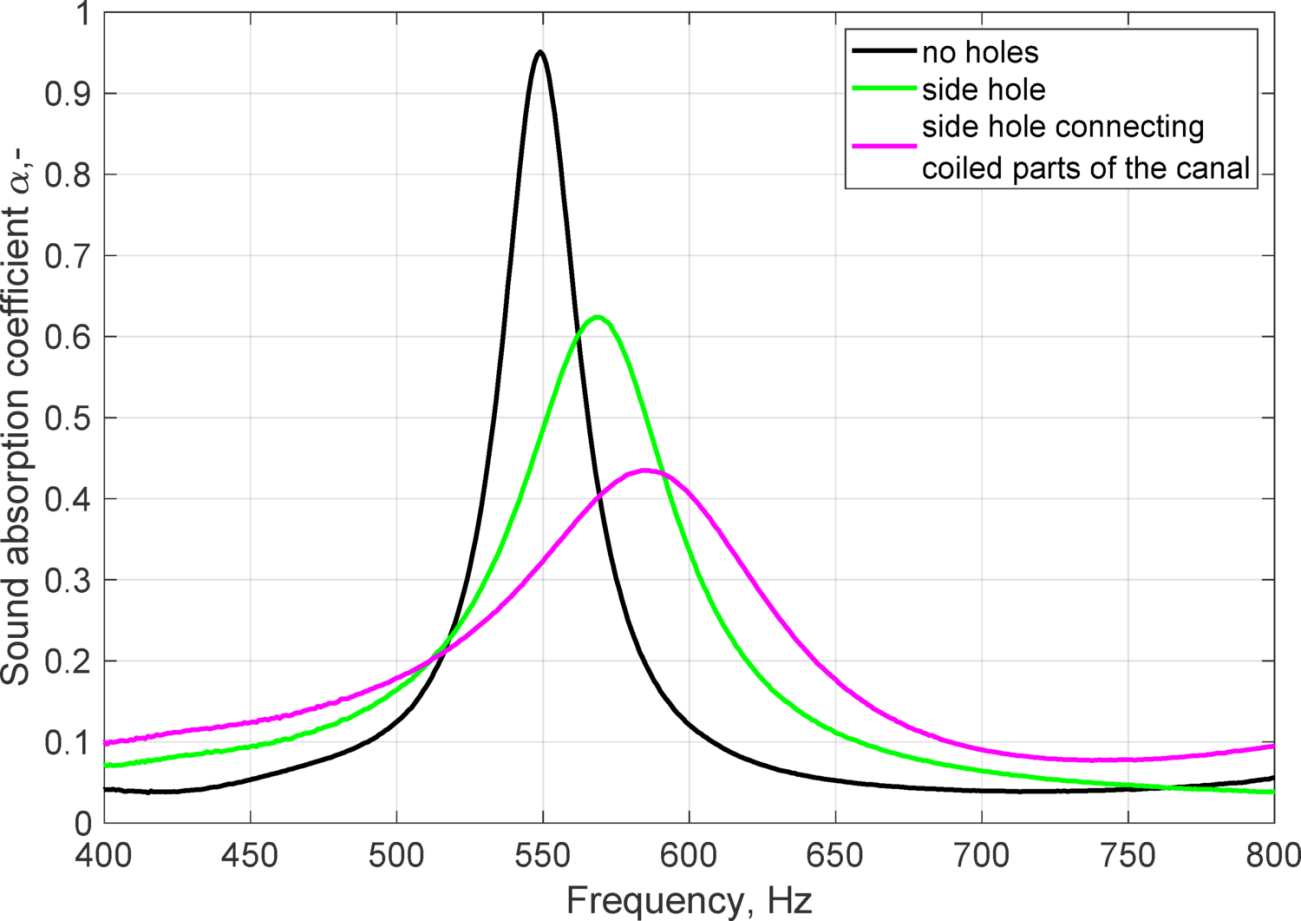
The sound absorption coefficient measured for the punctured sample.

To investigate the influence of ruptures in the canal walls caused by random events during printing, the sample was deliberately punctured with a tungsten wire 0.6 mm in diameter. The authors investigated the feasibility of employing FEM simulations for analyzing printing defects and microslits. However, due to the complex and highly variable nature of the infill structure within the samples, it is currently infeasible to perform precise simulations. Therefore, the experimental method remains the most reliable scientific approach for this study. The sample chosen for the experiment was printed with a PET-G filament (diameter of 0.4 mm, layer height of 0.2 mm). Two variants were tested: first, the sample was punctured once from the side to reach the canal. Then, the hole was lengthened to make a connection between the coiled parts of the canal (Fig. 7). The punctured sample was prepared to artificially simulate defects that may occur during the typical 3D printing process, as described in the previous section and illustrated in Fig. 5. In the experimental study, it was crucial to first measure a properly printed sample without microslits and then introduce artificial defects to systematically investigate their impact on the sound absorption properties of the metamaterial. The sound absorption coefficient of each variant was tested, and the results are shown in Fig. 8.

The results of the sound absorption coefficient measurements showed that the continuity of the canal walls is of crucial importance for the performance of the sample. Even for small ruptures less than one millimeter in diameter, the sound absorption coefficient decreases. Such small changes in the geometry cause mismatching of the impedances and shift the resonance frequency, decrease the maximum sound absorption coefficient by half, and decrease the quality factor of the resonance significantly. The observed effect is similar to what was previously observed in Fig. 6 for the samples with no obvious defects; some random events during printing may have most likely caused the rupturing of the walls of the coiled parts of the canal, changing its effective length and affecting the overall performance of the samples.

## Discussion

This paper concerns the issues of 3D-printed metamaterial unit cells with a coiled-up Helmholtz resonator. Three main points are discussed: the general approach for printing and sealing measurement samples with a coiled-up resonator, the choice of the 3D printing technique (FDM, SLS, DLP), and the parameters of the FDM settings. For the geometry described in the present research, a coiled-up Helmholtz resonator, it is not always possible to print a designed measurement sample in one piece, particularly when using SLS or DLP printing technology. For this reason, the first topic discussed in this research involved sealing the halves of the sample to match the original designs. The best printing parameters available within the research were used for the fabrication of the samples. Three different ideas were tested: joining the halves with six screws with no additional sealing, using a ring seal, and using a shaped seal (together with the six screws). It was shown that, no matter the printing technology or the filament type, neither of the proposed solutions resulted in a satisfactory sound absorption coefficient. Although the connection seemed tight, some microslits must have been formed, resulting in increased additional sound absorption inside the canals and, as a result, a decrease in the measured sound absorption coefficient. This effect was previously attributed to the surface roughness caused by the printing parameters^25^; however, after printing the measurement samples in one part, we now know that the surface roughness can cause only a slight decrease in the sound absorption coefficient (from 0.99 in the model to 0.92–0.95 in the measurements, irrelevant from a practical point of view), and the mismatching of the impedances is caused mainly by the additional sound absorption of the microslits at the connection of the halves. The only way to obtain reliable results is to print the samples in one part, without the need for joining the halves. Importantly, similar results could be observed for all the tested printing technologies. Considering the costs and feasibility of these printing techniques, FDM seems to be the most advantageous technique. To complement the findings of previous studies by the other authors, the FDM printing settings and the choice of filament were also investigated for the samples printed in one. All the tested parameter combinations (nozzle diameter, layer height) were generally worse than the combinations indicated as required by the other authors. Rather than ensuring high precision of the printout, preventing random printing defects, which may occur regardless of the printer settings, is more important. One such defect resulting in connecting coiled parts of the canal was deliberately recreated and was shown to be crucial for the acoustic performance of the sample. These findings indicate that the results previously attributed to filament choice or infill ratio might have been caused by random defects, and it should be emphasized that avoiding random printing defects is more significant than the choice of filament or printing parameters. Therefore, there is no need for a detailed focus on the exact printing method or printer configuration, as essentially all the tested technologies allow good acoustic performance of the samples to be achieved, provided that the postprint quality is verified since random printing errors are unavoidable regardless of the 3D printing technique or printer settings.

## Methods

### Numerical model

A numerical model of the metamaterial was created in COMSOL Multiphysics 5.6, simulating the sound absorption coefficient measurement setup in an impedance tube. The modeled sample was placed at the end of the cylindrical waveguide with a 10 cm diameter, and a hard wall boundary condition was applied to the sidewalls of the tube. At one end of the tube, a combination of background pressure field excitation and plane wave radiation conditions was simulated, and at the other end, the model of air inside the metamaterial was placed. The reflection coefficient was determined as the ratio of the reflected sound pressure to the background sound pressure averaged over the tube cross-sectional surface. The metamaterial structure was simulated using a narrow-region acoustic physics interface, which accounts for viscous and thermal losses within the model. In the case of simple geometries, using a narrow region acoustics model has a significant advantage over more detailed thermoviscous acoustics modeling, as no boundary layers are needed. This approach greatly reduces the mesh size and computation time with a marginal loss of accuracy. The model was meshed using a tetrahedral mesh with at least six elements per shortest wavelength; additionally, smaller elements were used in the vicinity of the resonator neck. The metamaterial walls were simulated using hard wall boundary conditions, meaning that the material was assumed to be perfectly rigid, with no surface roughness. The simulations were performed using a frequency domain study with 24 frequencies per octave. The model setup was validated by simulating simple, noncoiled Helmholtz resonators and comparing the resonant frequencies with analytical models, as well as simulating one of the metamaterial structures described in the study by Huang et al.^9^ and obtain a good match of resonant frequencies.

### Measurements

The sound absorption coefficients of the samples were measured in an impedance tube for the normal incidence angle of a sound wave. The measurements were taken according to the transfer function method described in the ISO 10534-2 standard^26^. The measurement setup consisted of a Brüel & Kjær 4207T impedance tube with two ¼’’ B&K 4187, a B&K 2716-C amplifier, a B&K 3160 sound analyzer, and PULSE software. The frequency resolution of the measurements was set to 1 Hz, and the measurements were taken within the range of 50–2000 Hz, determined by the diameter of the sample – 100 mm.

### 3D printing details

#### FDM printer and parameters

The FDM printer used to fabricate the measured samples was based on Tevo Tarantula Pro and was modified mainly via the installation of a dual-drive direct extruder and TMC2209 stepper motor controllers. This approach allowed us to enhance the quality of the printouts since direct extruders tend to have fewer feed inaccuracies and eliminate the slip generated by the Bowden tube. On the other hand, the TMC2209 stepper motor controllers enable displacement along the cartesian axes with 1/265 microstep interpolation, as opposed to the 1/16 or 1/32 interpolation offered as a standard in desktop 3D printers. A smaller number of microsteps allows smoother surfaces to be obtained, especially when printing circular shapes.

For printing sealings (both ring seal and shaped seal), the following settings were used:

- The nozzle diameter is 0.5 mm,
- 24 perimeters of the vertical shell.
- The printing speed was 15 mm/s.
- The head temperature was 245 °C.
- The bed temperature was 65 °C.
- No retraction,
- No infill,
- No top or bottom solid layers;
- Avoid perimeter crossing setting - on,
- Ironing–on, 5 mm/s; flow rate, 3%; 0.1 mm overlap.

This set of parameters was used to ensure minimal roughness of the sealing surfaces and therefore increase the sealing effectiveness.

#### DLP printer and parameters

In this study, photocured resin samples were produced using a standard Elegoo Saturn machine. The parameters of the printout were as follows:

- Material type: standard UV 405 nm resin,
- Layer height: 0.05 mm.
- Number of bottom layers: 6.
- Bottom layers curing time: 18 s,
- Normal layer curing time: 1.9 s.

The samples were oriented flat on the buildplate. Typically, a high coverage of flat and continuous areas causes high suction in the FEP film; in this study, the buildplate increase speed was set to 30 mm/s, and the increase height was set to 10 mm to allow the resin to flow uniformly between the cured layer and the next layer.

#### SLS printer and parameters

The machine used to fabricate the specimens in the present study was Sinterit Lisa Pro Rev. E. with its proprietary slicer Sinterit Studio. Most of the commercially available SLS 3D printers do not allow for adjusting many of the settings; thus, the samples were printed with the following parameters:

- Powder type: PA12 (Polyamide) SmoothLayer height: 0.125 mm.
- Laser power ratio: 0.95.
- Layer height: 0.125 mm,
- Orientation: flat, vertical and inclined.

## Data Availability

The datasets used and analyzed during the current study are available from the corresponding author upon reasonable request.
